# Computer-Assessed Preference-Based Quality of Life in Patients with Spinal Cord Injury

**DOI:** 10.1155/2017/4543610

**Published:** 2017-08-30

**Authors:** Enea Parimbelli, Caterina Pistarini, Gabriella Fizzotti, Carla Rognoni, Giampiero Olivieri, Silvana Quaglini

**Affiliations:** ^1^Department of Electrical, Computer and Biomedical Engineering, University of Pavia, Pavia, Italy; ^2^ICS Maugeri, Nervi, Italy; ^3^ICS Maugeri, Pavia, Italy; ^4^CERGAS, Bocconi University, Milan, Italy

## Abstract

**Objectives:**

Our aims were to (1) measure quality of life (QoL) in spinal cord injury (SCI) patients using different methods and analyze differences; (2) enable targeted treatments by identifying variables that affect QoL; and (3) provide decision-makers with useful data for cost-utility analyses in SCI population.

**Methods:**

Seventy-one participants were enrolled. The computer-based tool UceWeb was used to elicit QoL in terms of utility coefficients, through the standard gamble, time trade-off, and rating scale methods. The SF36 questionnaire was also administered. Statistical analyses were performed to find predictors of QoL among collected variables.

**Results:**

Median values for rating scale, time trade-off, and standard gamble were 0.60, 0.82, and 0.85, respectively. All scales were significantly correlated. Rating scale and SF36 provided similar values, significantly lower than the other methods. Impairment level, male gender, older age, living alone, and higher education were correlated with lower QoL but accounted for only 20% of the variation in utility coefficients.

**Conclusions:**

Demographic and clinical variables are useful to predict QoL but do not completely capture utility coefficients variability. Therefore, direct preference-based utility elicitation should be strengthened. Finally, this is the first study providing data that can be used as a reference for cost-utility analyses in the Italian SCI population.

## 1. Introduction

Regular measurements of quality of life (QoL) during follow-up of patients with chronic conditions help to obtain a global evaluation of a treatment effect, to detect new problems, or, more generally, to early detect a change in health conditions. This is even more important for those patients, like persons with spinal cord injury (SCI), whose QoL may be affected by physical deficits, psychological and socioeconomic problems, or difficulty reintegrating into employment or education. One possibility of increasing QoL in these patients is to understand which health problems are more correlated to QoL, in order to plan focused interventions to alleviate such problems. Interventions that target these dimensions in order to improve well-being and QoL through rehabilitation plans have proven to be effective in helping patients [1, 2]. Moreover, it could be interesting to investigate if different phases of SCI and/or specific social and familiar conditions are correlated to different QoL. All the information is important to build an individual rehabilitation plan.

In addition to their exploitation at an individual patient's level, QoL measurements are more and more used at a population level. As a matter of fact, options that may impact a population health, such as introducing a new diagnostic or treatment strategy, often undergo an economic evaluation to inform decision about their implementation. Most of those cost-effectiveness analyses consider as their primary endpoint not only life duration, but also its quality, and for this reason they are more appropriately called “cost-utility” analyses. Their combined endpoint is in general represented by Quality-Adjusted Life Years (QALYs). Supposing that the life of an individual can be divided into a set of time intervals, according to different health states that the individual may experiment, QALYs are calculated as the weighted sum of those time intervals (expressed in years) multiplied by the so-called “utility-coefficient” (UC). UCs range from 0 to 1 (0 representing death and 1 perfect health) and are a measure of the desirability of a health state [3].

There are different methods for measuring QoL. The most appropriate one is represented by* direct preference-based methods* that directly provide UCs. They are called so because, during a face-to-face interview, the patient must express a preference between different options. For example, using the standard gamble method, the patient is asked to choose, within a hypothetical scenario, between living the rest of his/her life in the health state that is being evaluated or playing a gamble which could result in complete recovery with probability *p* or sudden, painless death with probability 1 − *p*. The assumption is that the lower *p* is, the more a patient experiences a poor quality of life. The probability *p* is varied until the patient is indifferent between the two choices. That value of *p* is taken as the UC of the state. Another example is the time trade-off method, where the patient is asked to choose between living his entire remaining life (*t*1) in the health state being evaluated or to live a shorter time (*t*2), but in a perfect health. The amount of time a patient is proposed to give up is varied until the patient is indifferent between the two choices. The more a patient is experiencing poor QoL, the higher amount of time he would give up to live in perfect health. The UC is then calculated as *t*2/*t*1. Not all the subjects are able to understand the rationale of those methods and consequently respond knowledgeably. For this reason, another direct method is often applied, namely, the rating scale method. It consists in a visual-analog scale, usually ranging 0–100, where the patient is asked to position the health state under evaluation. Although it is easier to use and useful for ranking health states in terms of desirability, it is not preference-based and normalized rating scale values are not suitable for QALY calculation [4–6].

A second category of methods for measuring QoL is represented by questionnaires. There exist both generic questionnaires, that is, not conceived for a specific condition, and condition-specific questionnaires. In general, they are composed of a set of multiple choice questions (frequently answered on a Likert scale) organized in sections referring to various aspects of the patient's life (multiattribute questionnaires). Some of them measure only the health-related quality of life, while others consider additional aspects affecting a person's well-being, such as social, economic, and family aspects [7]. Each instrument may have a different algorithm to calculate overall scores by combining the partial scores of each section. Popular examples of generic questionnaires are EuroQol, Short Form 36 (SF36) [8], and Quality of Life Index (QLI) [9]. Disease-specific questionnaires may be either developed ad hoc for the disease or adapted from the generic versions. For example, a modified version of the SF36 questionnaire [10] has been developed for patients who need a wheelchair for moving around. In the modified version, questions about physical functionality have been rephrased accordingly. Also QLI offers a modified version for spinal cord injury where questions about the most common impairments caused by SCI have been added to the standard questionnaire (e.g., about the ability to go places outside home, the ability to have children, or the ability to clear lungs).

Questionnaires are simpler to administer compared to preference-based methods, so that some can be self-administered or administered by telephone [11]. However, they have some drawbacks. First, they detect change in symptoms and functions, but not in how these are valued by the individual patient [12]. Moreover, evidence suggests [13] that statistical models embedding demographic, economic, social, and clinical information often do not allow us to completely capture the variability registered using preference-based scores. In addition to that, the availability of multiple questionnaires raises questions about their relative merits [14] and often generates difficulties in comparing results obtained with different instruments [15]. Specifically about SCI, recent studies have highlighted the paucity of evidence regarding measurement properties in this condition [16] and the numerous attempts to add, delete, or modify items in SF36 have resulted in a large number of variants, often with minimal supportive psychometric evidence [17]. Some of these variants, for instance, SF-6D, have been suggested to be more appropriate for the specific physically impaired SCI population [18].

Finally, to be used in cost/utility analyses, a questionnaire score must be converted to a UC. To this purpose, algorithms have been developed for some questionnaires (that for this, reasons can be viewed as* indirect preference-based methods* for utility elicitation). In those cases, UCs are obtained by a model previously assessed by fitting the questionnaire scores on standard gamble or time trade-off UCs collected in the same sample of patients. Such models are available for EuroQol and SF36 (and its reduced version SF12). Nevertheless, as we can read in the EuroQol website [19], many studies that directly elicit preferences from general population samples are still under development and their results will take relevant time to be disseminated. Until the results of such studies would become available in the form of value sets for the EQ-5D-5L, the conversion of EuroQol scores into UCs would be implicitly biased by the characteristics of the limited population on which the conversion algorithms have been developed. Moreover, Pickard et al. [20] showed that UC calculated from SF36 and SF12 with different algorithms produced a wide range of incremental cost/utility ratios (ICURs) that could potentially lead to different reimbursement decisions.

The aim of this work is threefold: first, to assess the associations and agreement between various direct preference-based methods and questionnaires; second, to understand factors that correlate with QoL, which helps focusing on specific aspects during rehabilitation to maximize its benefits; and third, to provide reference values for UCs in the SCI Italian population, in order to enable the scientific community to use them in further cost/utility studies.

## 2. Materials and Methods

### 2.1. Subjects

Seventy-one participants, 42 males and 29 females, with a mean age of 55 ± 16 years, were recruited from October 2014 to December 2015 among hospitalized patients in IRCCS Fondazione Salvatore Maugeri Hospital in Pavia, Italy. Informed consent was obtained from all participants and the study has been approved by the hospital ethical committee (protocol number 2064 CE). The sample included 17 individuals at their first hospitalization after the injury (i.e., acute patients) and 54 participants who were hospitalized for a few days to undergo a planned rehabilitation session (i.e., chronic patients). Participants were further characterized by a set of demographic information consisting of sex, occupation, education level, and marital status. Injuries were classified following the American Spinal Injury Association Impairment Scale (AIS) classification [21]. The AIS scale grades patients based on their functional impairment as a result of the injury, from A (complete lesion, no sensory or motor function is preserved in the sacral segments) to E (normal, for patients who completely recovered after a SCI). Additional clinical assessment included date, cause, and vertebral level of the injury; tetraplegic/paraplegic/tetraparetic/paraparetic functional status of the patient; bladder and bowel functional assessment; presence of chronic comorbidities (hypertension, diabetes, and anxiety/depression); and pain, measured through the visual-analog scale [22] (pain VAS, range 0–10). We also collected information on three widely adopted functional scales that measure the independence of SCI patients, namely, the Italian version of the Spinal Cord Independence Measure (iSCIM, range 0–100) [23, 24], the Walking Index for Spinal Cord Injury (WISCI, range 0–20) [25], and the 10-meter walking test (TWT) [26]. The reason behind is that we found evidence in the recent literature that the degree of independence in walking and self-care [27, 28] and the level of pain experienced by participants [29, 30] have a relevant effect on QoL of patients with SCI.  Table 1 presents a summary of the characteristics of the participants of our study.

### 2.2. Instruments

**Figure 1:**
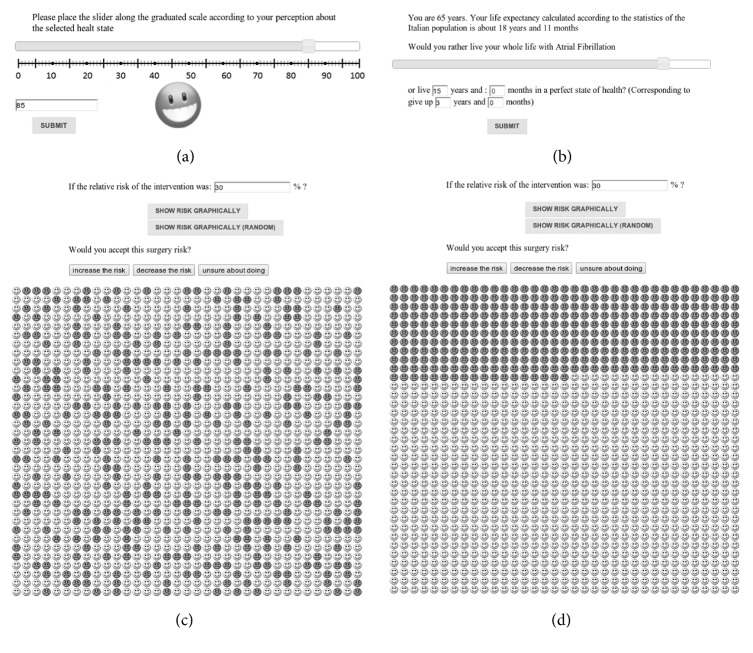
The user interface that UceWeb provides for eliciting utility coefficients using rating scale (a), time trade-off (b), and standard gamble ((c) with random smile arrangements and (d) with sequential arrangement). Visual aids, like sad, dark/happy, light smiles, represent the risk percentage and facilitate answering the elicitation questions for patient and interviewer [31].

We elicited UCs using UceWeb, a computerized tool we developed during the last few years [31, 32]. UceWeb implements the rating scale, standard gamble, time trade-off and its daily time trade-off variant, and willingness-to-pay methods (only the first three methods have been used in this work) and supports patient and interviewer in a user-friendly elicitation process which minimizes variability in the way the different methods are administered.  Figure 1 shows the graphical user interface of the tool.

We took advantage of the presence of hospitalized participants to perform the elicitation during a visit with their assigned physician (previously trained on the use of utility elicitation methods). This also allowed collecting feedback from the interviewer about the elicitation methods using a simple scale ranging from 0 to 4 (“how would you define the degree of understanding of the elicitation method?” 0: patient did not understand at all, 1: low, 2: sufficient, 3: good, and 4: perfect) and having participants assisted by an interviewer that they already knew and trusted.

As already mentioned, QoL in the same participants has also been assessed using a paper-based SF36 questionnaire, the scores of which were converted to UCs using Brazier et al.'s formula [33] to enable direct comparison with preference-based measures.

### 2.3. Analysis

After testing the distributions of UCs for normality using a Shapiro-Wilk test, we decided to use nonparametric measures as median and quartiles for descriptive statistics and Spearman coefficient for correlation analysis. Number of floor and ceiling values and number of missing values were calculated for each of the preference-based scores. The Mann–Whitney test was used to assess significant differences among variables, with a priori set to *p* < 0.05 for all reported analyses. A robust linear regression [34], employing M-estimators, was used to perform multivariate analysis. A stepwise variable selection procedure based on the Akaike Information Criterion was used to perform feature selection on the multivariate regression model. The R software [35] was used for all the statistical analyses.

## 3. Results

All participants reported a good understanding of elicitation methods in the feedback questionnaire, where the average score was greater than 3.1 (where 3 = good understanding and 4 = perfect understanding) for all the methods. This was also reflected by the low number of missing values for the UCs, which was limited to 3 participants not being able to complete the elicitation using standard gamble.

Median and interquartile range values for rating scale, time trade-off, and standard gamble were 0.60 (0.50–0.80), 0.82 (0.57–1), and 0.85 (0.6–1), respectively. Considering that the SCI population in Italy is about 70,000 individuals [36] and considering our sample size of 71 participants, those values are estimated with an error margin of about 11%. These data can be directly used in the calculation of QALYs in cost-utility analyses that, as we mention in the introduction, rely on UCs to correct life expectancy depending on its quality. To our best knowledge, this is the first study reporting UCs for an Italian SCI population and, for this reason, we believe that such data would be useful to enhance model quantifications in incoming cost/utility studies assessing interventions for SCI in Italy.

**Figure 2:**
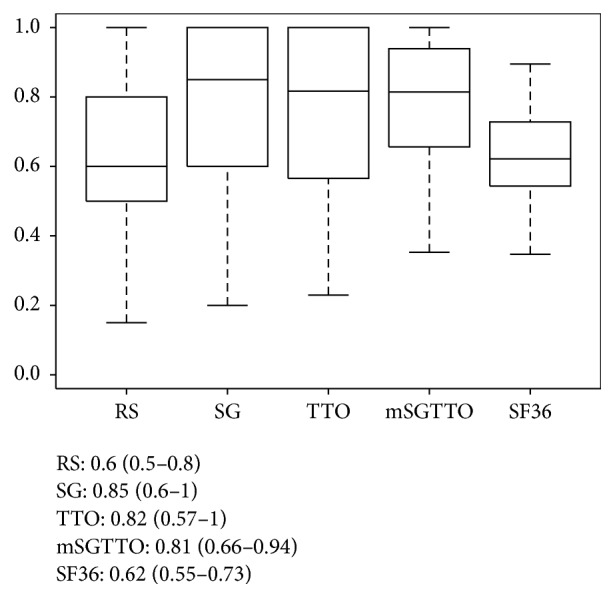
Boxplot of the utility coefficients obtained with rating scale (RS), standard gamble (SG), time trade-off (TTO), mean of standard gamble and time trade-off (mSGTTO), and SF36 questionnaire score converted to utility. Median (25th and 75th percentile) is also reported.

Figure 2 summarizes the values of collected UCs. Rating scale values were similar to SF36 and significantly lower (Mann–Whitney test, *p* < 0.001) than those obtained using time trade-off and standard gamble. As illustrated in Figure 2, those methods provided a number of UC values equal to 1. This also partially explains the higher degree of dispersion of those UCs with respect to SF36.

As we already reported in previous works exploiting UceWeb [31, 37], standard gamble and time trade-off scores are similar but of course not identical, and the mean of their values seems to better describe the overall QoL than considering each of the scores separately. For these reasons, we chose to run our subsequent analyses also including the mean of standard gamble and time trade-off UCs (mSGTTO).

**Figure 3:**
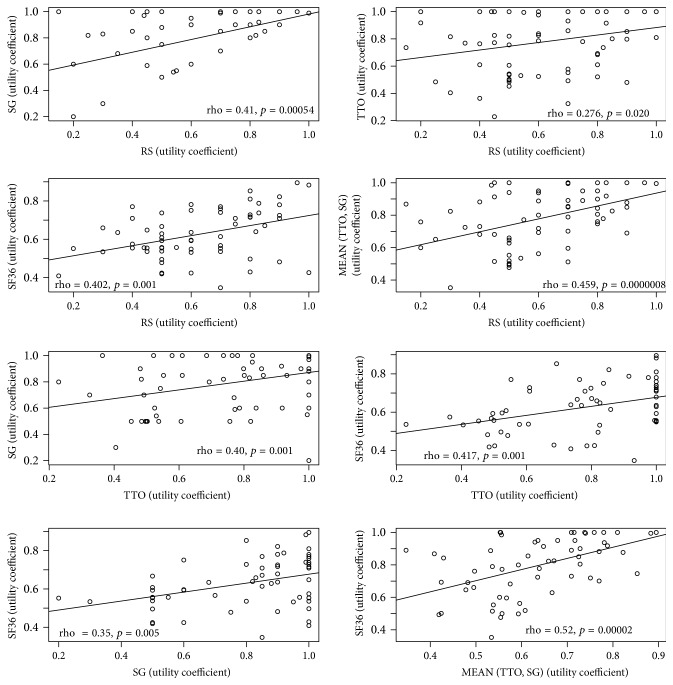
Correlation plots of the utility coefficients obtained with the different elicitation methods. Rating scale (RS), standard gamble (SG), time trade-off (TTO), mean of standard gamble and time trade-off (mSGTTO), and SF36. Higher values of UCs correspond to better quality of life (0: death, 1: perfect health). Rho coefficients and corresponding *p* values are presented for each pairwise correlation.

As reported in Figure 3, all scales were significantly correlated. Interestingly the best correlation was found between the mTTOSG and the UC derived from SF36 (rho = 0.52, *p* value = 0.00002).

*Impact of Patient Characteristics on Quality of Life Variables*. As mentioned, we were interested in finding which patient characteristics may explain the differences in QoL experienced by the participants.  Table 2 reports the *p* values of the Mann–Whitney test for difference in the observed values of mTTOSG when grouping our study population according to categorical characteristics of the participants. None of these are significant when the entire group of study participants is considered. However, some more local effects are visible on specific subpopulations and specific elicitation methods.

**Figure 4:**
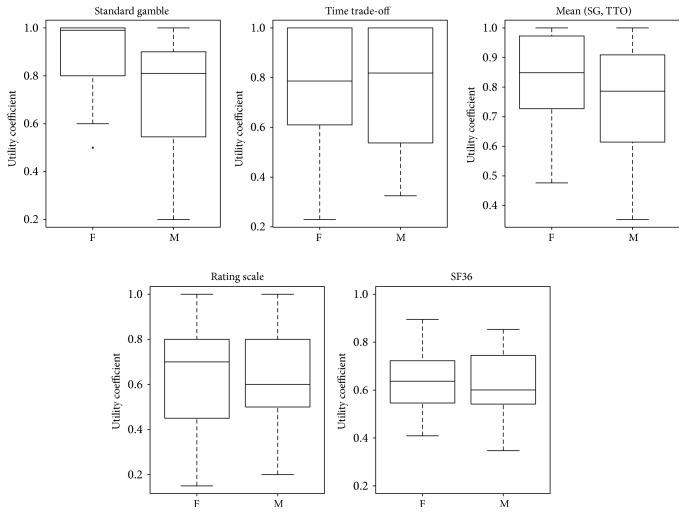
Utility coefficients elicited using standard gamble show significantly lower results for males. The circle represents the outlier.

A first example regards the effect of sex on UCs elicited using standard gamble.  Figure 4 shows that standard gamble is the only elicitation method which highlights differences between males and females (Mann–Whitney test *p* value = 0.0066).

Regarding AIS classification, note that none of our subjects had less severe lesions than AIS C (26 A, 15 B, and 30 C). The only difference we found in QoL is within patients in chronic phase, where AIS C participants have higher UCs than classes A and B.

No significant difference was observed in the QoL of tetraplegic and paraplegic participants (Mann–Whitney test, *p* value = 0.11). Similarly, cause of lesion was not directly related to QoL (*p* value = 0.35), while the presence of neurogenic bowel was associated with lower UCs (*p* value = 0.0035), when measured by SF36.

Table 3 shows the results of the multivariate analysis where mTTOSG score is the dependent variable. Increasing age of the patient had a rather small negative effect on preference-based QoL. More precisely, an increase of 10 years in the age of the patient would lead to a decrease of 6% in the mTTOSG score. Other significant independent variables were education level (negative effect), marital status (positive effect), and AIS classification (positive effect of AIS C).

Regarding the correlation between QoL and functional scales for independence, at univariate analysis none of the scores was significantly correlated with mSGTTO with the best results obtained for iSCIM (*p* value 0.095). Moreover, given that assessment of these scores is not routinely performed in the FSM hospital, data were very sparse and only eight participants had complete data for all the four scores. Nonetheless, a multivariate robust linear regression analysis including only the four variables showed that WISCI, TWT, and pain VAS are significantly correlated to mTTOSG.

## 4. Discussion and Conclusion

In this paper, we have reported on a study where preference-based QoL measures were collected for 71 SCI patients. We used a computer assisted tool to collect such data, in the form of utility coefficients, using direct elicitation methods. Standard gamble and time trade-off methods provided values that were significantly lower than those provided by rating scale and SF36. This finding is coherent with similar results obtained in other clinical domains [38]. This is probably an effect of introducing an actual choice between alternatives in the elicitation process (i.e., facing a risk of death in standard gamble or trading some of the available time in time trade-off) that translates to an increased ceiling effect (upward bias) for time trade-off and standard gamble [39]. The latter shows this effect mostly in female patients. As known, in this method the patient is asked if s/he would accept a hypothetical gamble resulting in death with a given probability *p* or in complete healing with probability 1 − *p*. Given the nature of the question, the difference between genders might be explained by the fact that males tend to be more risk-prone than females [40], leading to lower values of utility. This is furtherly sustained by the fact that, in our specific patient population, the most frequent (40% of the cases) causes of injury among male participants were traumas like road or sports accidents, while the leading cause for women was canal narrowing (e.g., due to disk degeneration, arthritis, or cancer).

In agreement with past literature [41–43], no significant difference was observed in the QoL of tetraplegic and paraplegic participants or in the QoL of patients with different cause of lesion.

Our multiple regression analysis highlighted educational level, marital status, and AIS as independent predictors of QoL. Participants with a lower education level (middle school or below) tend to have higher UCs. This might be due to the higher adaptation capabilities of individuals with low education when compared to people who have invested significant efforts in education and now realize that they are not able to reap the benefits anymore. As a matter of fact, disability acquired from SCI might result in abrupt change of a person social and employment status (63% of the participants considered were unemployed at the time of the analysis) which is more difficult to accept for individuals with more ambitious goals [44]. Participants who are not married also have lower QoL probably due to the fact that they lack the emotional and physical support of a close relative living with them [45]. Finally, participants classified with AIS C had a better UC than ones with more severe lesions, also due to their significantly higher chance of transitioning to less severe D and even E levels [46, 47]. However, this finding has been observed in chronic patients only. This might be explained by the fact that patients in acute phase are still hospitalized and thus living in a* protected* environment since the injury event. In such a situation where specialized assistance is guaranteed by hospital personnel at all times, the increased disability of ASIA A and B patients might not have the same relevance as in the home setting. On the contrary, chronic patients have already experienced the disability burden at home, so that they are more aware of possible negative consequences.

A slightly different finding with respect to the literature concerns the role of neurogenic bladder and bowel management. Although those conditions are given importance by both clinicians and patients [48, 49], our data did not highlight a significant correlation with QoL. A previously published work [41] suggested that these complications were associated with lower SF36 scores. A similar result was indeed obtained for our population but only between SF36 UC and neurogenic bowel.

About the relationships among UCs measured with different methods, statistically significant correlations were found between SF36 scores and the other UCs, but the best correlation coefficient barely exceeded 0.5, and true linear correlation was only observed for higher UC values. The statistical analysis highlighted the fact that the set of demographic and clinical data we collected only partially explain the high variability in the perceived QoL.

These findings suggest that clinicians not only should rely on the routinely collected data or questionnaires to infer QoL of their patients but should also include direct preference-based utility elicitation, especially when they plan to perform economical evaluations such as cost-utility analyses in their studies. To this extent, our aim of providing reference values for UCs in the Italian SCI population is of particular interest. As known, the context of life significantly affects the perception of QoL [50]. For this reason, UCs may be very different from country to country. This is also supported by the fact that the community developing EuroQol, the most widely used instrument for QoL assessment, has been committed for a long time to increase the number of countries involved in valuation studies that produce country-specific value sets [51] to be used in economic evaluation of healthcare programs.

We argue that even deeper analyses might be informative to cost-utility studies. In this initial effort, we provided overall UCs for the Italian population in general, because splitting the sample according to patient characteristics would result in too small subsamples. However, our intention for future work is to provide facilities to support subpopulation analysis within the same country [52]. Since additional elicitations would be needed in order to increase the sample size, we point out that a demo version of the UceWeb tool is available at http://labmedinfo.org:8194/UceWeb for all the researchers interested in this area, which allows collecting UCs for any health state, after profiling patients according to age, gender, marital status, educational level, and ethnic and geographic origin.

To our best knowledge, only a few studies report UCs for SCI population. Two works from Lin and colleagues [53, 54] report using standard gamble and time trade-off methods, administered through telephone interviews, for the assessment of QoL in SCI patients. Another work from Lee and colleagues [18] used Brazier's algorithm to convert SF36 scores to UCs. Interestingly, the authors found that those UCs were able to capture significant variations of QoL at follow-up [18] for patients that developed a urinary trait infection. This is in agreement with our finding suggesting UCs as an appropriate measure of QoL in SCI. However, this result may be difficult to generalize, since as already mentioned [20], UCs derived from questionnaires rather than direct utility elicitation may be affected by high variability according to the conversion algorithm used.

As a review from Ku [55] and several other works [56–60] pointed out, health-related quality of life instruments, and especially SF36, has been widely used to quantify the effect of SCI on QoL. However, previous meta-analyses from Dijkers [50, 61] highlight how these instruments inevitably consider a limited set of predefined dimensions (usually health-related) and in particular are only sensitive to the “objective” evaluation of QoL (e.g., measuring independence or functional status) while being much less sensitive to subjective evaluation and individual expectations and priorities [50, 62]. This furtherly motivated our research of utility elicitation through direct methods.

Our study has some limitations. First of all, we decided to set up the elicitation tool such as all the sessions had the same ordering of the methods, that is, rating scale first, time trade-off second, and standard gamble last. This was to ensure every participant was interviewed following the same procedure, thus minimizing interparticipant bias effect. However, different ordering of the methods might influence elicitation results themselves, pushing the UCs of the last elicitation towards higher values [63]. This is also visible in our results (see boxplots reported in Figure 2) where standard gamble utilities appear to be slightly higher than the ones elicited using time-trade-off, even if no significant difference is revealed by statistical tests (Wilcoxon signed rank test, *p* value = 0.43). In future work, a larger number of participants would be needed to effectively randomize the ordering of the methods and check for effects of different ordering choices on the values of the UCs. Secondly, besides the demographic variables we collected, also other less evident effects might influence UCs obtained using some of the methods. This makes isolating the net effect of SCI from other contingent, non-health-related factors, a difficult task. For example, a recent work highlights that the presence of children or “significant others” in the patient's family has an important influence on time trade-off and in general on all the methods where the patient is asked to face hypothetical scenarios involving death [64]. Third, our choice of using standard SF36 as a validation reference might have been improved by using the walk-wheel adaptation of the questionnaire [10].

Despite those limitations, we think our study represents an advance in the state of the art for what concerns QoL of individuals affected by SCI. First of all, we highlighted differences in the QoL measured by different methods and provided explanations for such differences. Moreover, we found some significant interesting correlations between UCs and some patient characteristics. This could help healthcare professionals in preparing more personalized treatment plans. Finally, we provided reference values for UCs in the Italian SCI population, which will be useful for carrying out cost/utility analyses in future economic evaluations of healthcare interventions addressing this condition.

## Figures and Tables

**Table 1:** Characteristics of the participants.

Category	Total (*N* = 71)
Median (range) age at interview (years)	59 (19–82)
Median (range) age at injury (years)	54 (0–75)
Sex	
Male *N* (%)	42 (59)
Female *N* (%)	29 (41)
Occupation	
Employed *N* (%)	14 (20)
Unemployed *N* (%)	57 (80)
Education	
Primary school *N* (%)	12 (17)
Secondary school *N* (%)	28 (40)
High school *N* (%)	25 (35)
University *N* (%)	6 (8)
Marital status	
Alone *N* (%)	29 (41)
Married *N* (%)	42 (59)
Level of injury	
Paraparetic *N* (%)	23 (33)
Paraplegic *N* (%)	27 (38)
Tetraparetic *N* (%)	8 (11)
Tetraplegic *N* (%)	13 (18)
AIS	
A *N* (%)	26 (37)
B *N* (%)	15 (21)
C *N* (%)	30 (42)
Phase	
Acute *N* (%)	17 (24)
Chronic *N* (%)	54 (76)
Median (range) number of comorbidities	1 (0–3)
Median (range) iSCIM	69 (10–100)
Median (range) WISCI	13 (0–20)
Median (range) TWT (sec)	20.5 (5–53)
Median (range) pain VAS	5 (0–10)

**Table 2:** Distribution of mTTOSG UCs in patients' groups split according to the different categorical variables.

Category	mTTOSG utility coefficient distribution: median (min, 25th percentile, 75th percentile, max)		*p* value^*∗*^
Sex	Male: 0.79 (0.35, 0.62, 0.76, 1)	Female: 0.85 (0.48, 0.73, 0.96, 1)	0.1566
Marital status	Married: 0.87 (0.50, 0.70, 0.94, 1)	Alone: 0.80 (0.35, 0.60, 0.91, 1)	0.3484
Education level	High: 0.73 (0.49, 0.65, 0.85, 1)	Low: 0.88 (0.35, 0.67, 0.97, 1)	0.08097
Employment	Employed: 0.79 (0.52, 0.72, 0.91, 1)	Unemployed: 0.82 (0.35, 0.63, 0.95, 1)	0.7845
AIS	A or B: 0.76 (0.35, 0.63, 0.89, 1)	C: 0.89 (0.48, 0.72, 0.95, 1)	0.07791
Level of injury	Tetraplegic: 0.72 (0.3527, 0.5298, 0.9009, 1)	Paraplegic: 0.83 (0.50, 0.69, 0.94, 1)	0.1072
Phase	Acute: 0.79 (0.52, 0.66, 0.90, 1)	Chronic: 0.83 (0.35, 0.66, 0.95, 1)	0.8704
Cause of injury	Trauma: 0.79 (0.35, 0.61, 0.93, 1)	Other: 0.85 (0.48, 0.73, 0.94, 1)	0.3546
Bladder function	Neurogenic: 0.78 (0.35, 0.64, 0.90, 1)	Normal: 0.90 (0.48, 0.72, 0.96, 1)	0.1336
Bowel function	Neurogenic: 0.79 (0.35, 0.62, 0.90, 1)	Normal: 0.87 (0.48, 0.71, 0.96, 1)	0.2946
Comorbidities	Yes: 0.77 (0.35, 0.62, 0.93, 1)	No: 0.84 (0.53, 0.76, 0.94, 1)	0.2187

^*∗*^Mann–Whitney test.

**Table 3:** Coefficients of the minimal robust linear regression model after selection of variables. ^*∗*^A binary dummy variable (AIS = C yes/no) for the AIS was used after the observation that chronic AIS C patients had higher utility coefficients than A and B.

Variable	Coefficient	Standard error	*p* value
(Intercept)	1.083	0.106	
Age	−0.006	0.0018	
Sex = M	−0.055	0.0432	
Education level			
High	0		
Low	0.104	0.0454	0.0260
Not married	−0.102	0.0464	0.0324
AIS^*∗*^			
AIS A or B	0		
AIS C	0.120	0.0502	0.0200
